# Glyphosate, a chelating agent—relevant for ecological risk assessment?

**DOI:** 10.1007/s11356-017-1080-1

**Published:** 2018-01-02

**Authors:** Martha Mertens, Sebastian Höss, Günter Neumann, Joshua Afzal, Wolfram Reichenbecher

**Affiliations:** 1 Institute for Biodiversity Network e.V. (ibn), Nußbergerstr. 6a, 93059 Regensburg, Germany; 2 Institute of Crop Science (340h), University of Hohenheim, 70599 Stuttgart, Germany; 3 Federal Agency for Nature Conservation (BfN), Konstantinstr. 110, 53179 Bonn, Germany

**Keywords:** Glyphosate, Chelating agent, GM crops, EPSPS, Mode of action, Risk assessment, Soil life, Nutrient availability

## Abstract

Glyphosate-based herbicides (GBHs), consisting of glyphosate and formulants, are the most frequently applied herbicides worldwide. The declared active ingredient glyphosate does not only inhibit the EPSPS but is also a chelating agent that binds macro- and micronutrients, essential for many plant processes and pathogen resistance. GBH treatment may thus impede uptake and availability of macro- and micronutrients in plants. The present study investigated whether this characteristic of glyphosate could contribute to adverse effects of GBH application in the environment and to human health. According to the results, it has not been fully elucidated whether the chelating activity of glyphosate contributes to the toxic effects on plants and potentially on plant–microorganism interactions, e.g., nitrogen fixation of leguminous plants. It is also still open whether the chelating property of glyphosate is involved in the toxic effects on organisms other than plants, described in many papers. By changing the availability of essential as well as toxic metals that are bound to soil particles, the herbicide might also impact soil life, although the occurrence of natural chelators with considerably higher chelating potentials makes an additional impact of glyphosate for most metals less likely. Further research should elucidate the role of glyphosate (and GBH) as a chelator, in particular, as this is a non-specific property potentially affecting many organisms and processes. In the process of reevaluation of glyphosate its chelating activity has hardly been discussed.

## Introduction

Glyphosate is a broad-spectrum herbicide ingredient active against both mono- and dicotyledonous plants. Glyphosate-based herbicides (GBHs) are the most widely used herbicides globally, due in large part to the cultivation of genetically modified glyphosate-resistant (GR) crops (e.g., soybean, corn, cotton, oilseed rape, and others) on millions of hectares worldwide (James 2017). The declared active ingredient glyphosate is not applied alone, but in combination with various formulants intended to increase its action. The use of GBH has increased significantly in the European Union, too, where GR crops are not authorized for cultivation. Here, the herbicide is applied pre-seeding, after harvest, for desiccation purposes (restricted in some countries), and in non-agricultural settings. Approval procedures for herbicide active ingredients such as glyphosate are regulated in the EU by specific legal frameworks^1^ that define also the requirements for reapproval. Glyphosate, authorized in the EU in 2002, has been in the process of reevaluation and the extension of its use, from the 15 December 2017^2^ for another 5 years, as proposed by the Commission has recently been approved by the member states.^3^ The European Chemicals Agency (ECHA), the competent EU agency for the assessment of dossiers for the classification of chemical substances, presented its final opinion (no classification for germ cell mutagenicity, carcinogenicity, and for developmental toxicity is warranted) in June 2017 (ECHA 2017).

Glyphosate is known to inhibit 5-enolpyruvylshikimate-3-phosphate synthase (EPSPS), an enzyme of the shikimic acid pathway that leads to the biosynthesis of aromatic acids phenylalanine, tryptophan, and tyrosine and a range of other substances. As this pathway is present only in plants and microorganisms, glyphosate was considered to be of low toxicity to humans and animals. More recent research, however, has raised concerns that glyphosate may be more harmful to animals and humans than previously expected (e.g., Myers et al. 2016). Glyphosate is also known as a potent chelator for minerals, a property that has been observed decades ago (Toy and Uhing 1964), even before the herbicidal effect of glyphosate was discovered (Komives and Schröder 2016). If glyphosate, in fact, binds essential minerals effectively, its application could lead to an undersupply of minerals that are essential co-factors in many biological processes in treated plants and potentially also in organisms feeding on such plants. This in turn could impact plant resistance to disease and affect human and animal health. Although the chelating properties are well known, this potential additional environmental risk was never adequately considered in the regulatory risk assessment (EFSA 2015a, 2015b).

While the chelating property of glyphosate was discovered and patented long ago, this literature review is performed to critically evaluate its role in effects that are not readily explained by the inhibition of EPSPS. To this end, not only peer-reviewed papers are considered, but also scientific opinions of EFSA on glyphosate (EFSA 2015a, 2015b) and on genetically modified GR crop plants (EFSA 2006, 2009, 2011, 2012).

### Properties and fate of glyphosate and GBH

Glyphosate (C_3_H_8_NO_5_P; N-(phosphonomethyl)glycine, MW 169) is a polar, water-soluble organic acid (given in acid equivalents a.e.). It is a potent chelator that easily binds divalent cations (e.g., Ca, Mg, Mn, Fe) and forms stable complexes (Toy and Uhing 1964; Cakmak et al. 2009). Glyphosate is used as salt, termed the active ingredient (a.i.), mostly as isopropylamine (IPA) salt (MW 228), less frequently as trimesium salt (MW 245). The purity of technical grade glyphosate is generally above 90% (WHO 1994). Depending on the type of use, recommended application rates of glyphosate range from 0.21 to 4.2 kg a.i./ha and do not exceed 5.8 kg a.i./ha (Cerdeira and Duke 2006; WHO 1994). According to EFSA (2015b), the maximum cumulative application rate in the EU is set to 4.32 kg/ha glyphosate in any 12-month period.

The declared active ingredient glyphosate is used in formulations which usually contain formulants (e.g., surfactants) that facilitate penetration of the active ingredient through the waxy surfaces of the treated plants and increase its activity. For agricultural or other uses, many GBHs are on the market, produced by a number of companies. The products contain glyphosate in various concentrations of acid equivalent (the active moiety), ranging from 356 to 540 g a.e./L^4^ and different adjuvants and surfactants. The latter are, in general, not identified on product labels and can be claimed confidential business information. The Roundup product line (Monsanto) is the major and best known formulation that, as a surfactant, very often contains polyethoxylated tallow amine (POEA), a complex mixture of di-ethoxylates of unsaturated and saturated tallow amines characterized by their oxide/tallow amine ratio (typically 15% or less of the final formulation) (Tush and Meyer 2016). POEA are significantly more toxic to animals, e.g., amphibia, than glyphosate itself (Howe et al. 2004) and have been shown to be active principles of human cell toxicity (Mesnage et al. 2013). In hard water, the presence of polyvalent cations (e.g., Ca^2+^, Mg^2+^) can lead to the formation of insoluble metal complexes of glyphosate and thus reduce its herbicidal effect. Monsanto,^5^ therefore, recommends addition of ammonium sulfate, competing with glyphosate for free Ca^2+^, to be used with Roundup and making separate applications if foliar micronutrient sprays are used.

Glyphosate use has risen enormously within the last years, in particular in countries where genetically modified (GM) glyphosate-resistant (GR) crops are cultivated. In the USA, agricultural use of glyphosate rose from 1995 (the year before the first GR crop was grown) to 2014 9-fold to 113.4 million kg, whereas global agricultural use of glyphosate rose almost 15-fold to 747 million kg, with more than 50% accounted for by GR crops (Benbrook 2016). In light of the great number of GR crops that are authorized in various countries or in the pipeline, increased use of glyphosate is expected, potentially reaching globally 1000 million kg by 2023.^6^ Despite increasing problems with glyphosate-resistant weeds (Green 2016), it will most likely remain one of the most used herbicides.

Glyphosate is a systemic herbicide active ingredient that is rapidly absorbed by plant leaves and transported to distant plant organs, in particular meristematic tissues. Plant organs with high metabolic rates, e.g., shoot apices and root tips, are important sinks for glyphosate, with the latter having the ability to release it to soil (Gomes et al. 2014). In general, metabolization is limited in most plants (Hoagland and Duke 1982), although in leaves, stems, and seed of genetically modified GR soybean, its main metabolite aminomethylphosphonic acid (AMPA) has been found (Arregui et al. 2004; Duke 2011). “Yellow flash,” temporary chlorosis of newly emerging soybean leaves observed in GR soybean sprayed with Roundup WeatherMax, has been attributed to the rapid metabolism of glyphosate to phytotoxic AMPA (Duke et al. 2012).

Glyphosate reaches soil via direct application, washing off from plant surfaces by rain, via air-borne drift (Davidson et al. 2001), precipitation (Battaglin et al. 2014), and via root exudation (Neumann et al. 2006; Laitinen et al. 2007), or by decomposition of treated plant material (Locke et al. 2008). Glyphosate (and AMPA) concentrations in soil can reach levels in the mg/kg (ppm) range, e.g., in Argentina (Aparicio et al. 2013) and the EU (Silva et al. 2017). It may be taken up by plants and be an environmental source of glyphosate exposure for non-target plants (Gomes et al. 2014). In particular, plant residues of glyphosate-treated plants such as roots and harvest residues can bear an intoxication risk for subsequent crops, since accumulation of glyphosate in young growing root tissues leads to high levels of glyphosate that is subsequently released during microbial degradation of the plant residues and can be taken up by non-target plants via contact contamination (Tesfamariam et al. 2009).

The organic acid glyphosate is strongly bound on soil minerals, with sorption depending on types, contents, and crystallinity of minerals, pH, phosphate content, and organic matter (Borggard and Gimsing 2008). Soils that contain higher amounts of organic matter, clay, and Fe and Al oxides can adsorb more glyphosate (Barrett and McBride 2006; Wang et al. 2005; Laitinen et al. 2009) and potentially reduce its toxic activity on roots (Hensley et al. 1978). Phosphate fertilization may remobilize bound glyphosate by replacing it at binding sites in soil, thus increasing the risks for glyphosate leaching and for uptake by plants (Bott et al. 2011; Gimsing et al. 2007; Simonsen et al. 2008). This may be particularly important when phosphate is applied at higher concentrations, e.g., in fertilizer placement strategies.

The strong tendency of glyphosate to sorb on minerals through its functional groups may mobilize bound trace metals by chelation and sorbed anions, e.g., phosphate, by displacement. Several elements, in particular Cu, Al, and P, could be mobilized within the surface layer of soils that receive high glyphosate rates. After such treatment, soils containing elevated concentrations of heavy metals and phosphate showed increased leaching of Cu, Zn, Al, Ni, P, Si, and As, whereas in mineral and organic soils with normal background concentrations of heavy metals, no increased leaching was observed (Barrett and McBride 2006).

Glyphosate exhibits low to very high persistence in soil. Its degradation is mainly performed by microorganisms and takes place under both aerobic and anaerobic conditions, leading either to formation of its main metabolite AMPA and glyoxylate or to sarcosine and glycine (Borggard and Gimsing 2008). Degradation rates depend on soil parameters and temperature and can differ significantly from one soil to another, half-lives of more than 300 and up to 428 days may be reached, whereas AMPA degrades much more slowly (EFSA 2015a, 2015b; Borggard and Gimsing 2008). In cold climates with seasonally frozen soils, glyphosate can persist over the winter (Laitinen et al. 2006). In a Finnish study, 20 months after application, 19% of the applied glyphosate was still found in the plow layer, and the amount of AMPA detected represented about 48% of the parent molecule glyphosate (Laitinen et al. 2009). Formulants, e.g., POEA, can also persist in soils into the following growing season and potentially longer, with homologs with unsaturated tallow moieties being degraded more rapidly than homologs with saturated tallow moieties (Tush and Meyer 2016).

The substances glyphosate and AMPA reach aquatic systems, too, including groundwater (Sanchis et al. 2012), and can lead to surface water concentrations in the μg/L to mg/L range (WHO 2005; Battaglin et al. 2014). In European countries, glyphosate and AMPA levels of up to 370 and > 200 μg/L, respectively, have been detected in surface water, whereas in groundwater, levels of both substances higher than 0.1 μg/l have been found (EFSA 2015a). Tolerable glyphosate levels in drinking water differ significantly between countries. For drinking water, the US maximum contaminant level (MCL) of glyphosate is 700 μg/L, higher than for other pesticides,^7^ whereas the EU tolerable level for pesticides is, in general, 0.1 μg/L.^8^ The half-life of glyphosate in freshwater systems may be prolonged by higher sediment concentrations of chelating metals (e.g., Cu, Fe), due to the formation of metal-glyphosate complexes that reduce the bioavailability of glyphosate to microbial decomposers (Tsui and Chu 2008).

Food and feed produced from glyphosate-treated plants may contain glyphosate and AMPA residues that could affect humans and animals negatively (Bai and Ogbourne 2016). To protect humans from potentially toxic pesticide residues, maximum residue levels (MRLs) have been set. But, as Crop Life America puts it, MRLs are not, as is commonly thought, directly related to toxicity of the product, they rather reflect the residues arising from the use of the crop protection product as recommended on the label.^9^ In the EU, food MRLs for glyphosate range from 0.05 mg/kg (ppm) for most animal products to 20 mg/kg (e.g., soybean, sunflower, barley, oat).^10^ For the latter two crops, EFSA (2015a) proposed new MRLs of 30 mg/kg and for wheat and rye MRLs of 20 mg/kg (actual MRL 10 mg/kg). These proposals are potentially due to observed values of up to 21.4 mg/kg glyphosate for barley and oat and up to 17.5 mg/kg in wheat and rye grain (EFSA 2015b). US tolerances for glyphosate residues in food (e.g., 40 mg/kg in oilseeds) and in feed (up to 400 mg/kg in non-grass feed^11^) can be significantly higher. In 2013, tolerance levels for glyphosate residues in US soybean were raised from 20 to 40 mg/kg, coinciding with industry development of new GM varieties with stronger tolerance to glyphosate (Cuhra 2015).

In GR soybean, residue levels of up to 8.8 mg/kg for glyphosate and 10 mg/kg AMPA have been found (Bøhn et al. 2014) and total levels (glyphosate plus AMPA) can reach more than 50 mg/kg (Cuhra 2015). Glyphosate and AMPA residues in food and feed are stable for at least 2 to more than 3 years in the different matrix types, with up to 4 years for high starch content matrices (EFSA 2015a). In the body, glyphosate is distributed widely (e.g., bone, liver, and kidney), but supposed to be excreted within 7 days (EFSA 2015a). Excretion occurs via urine and feces (Acquavella et al. 2004; Krüger et al. 2013a, 2014; von Soosten et al. 2016). In 2012 and 2013, more than 50% of urine samples collected 2001–2015 from young humans in Germany contained glyphosate and AMPA concentrations at or above detection level (0.1 μg/L) (Conrad et al. 2017).

## Modes of action of glyphosate

### Impacts on plants

Early in vitro studies on glyphosate effects on microorganisms (e.g., *Escherichia coli*) and on plant cells and organs led to conflicting results regarding the mode of action. Various effects of glyphosate have been described, e.g., on synthesis of proteins, phenolic compounds, and chlorophyll, on photosynthesis, respiration, and metal ion chelation (Cole 1985). In their review on biochemical effects of glyphosate, Hoagland and Duke (1982) concluded that the rapid absorption and translocation in plant tissues and the lack of metabolism suggest alterations of a wide variety of functions and enzymes by glyphosate with multiple sites of action. Impacts on carbon metabolism, nitrogen metabolism, oxidative stress, and lignin production have been described (Gomes et al. 2014). The latter authors also stress that the role of the main metabolite AMPA in altering plant physiology is far from being clear. Hormetic effects of non-lethal doses of glyphosate (acting as a growth regulator) on non-resistant plants have been observed, too. This means that low rates (in general, up to 25 g a.e./ha, varying between species and development stages) can induce shikimic acid accumulation and increase crop plant growth, photosynthesis, stomatal opening, and seed production, but how these effects are brought about is not clear (Brito et al. 2017). For practical uses, induction of hormesis by glyphosate is not very reliable (Belz and Leberle 2012).

The target mode of action of glyphosate is its toxic activity against any plant. As shown by Steinrücken and Amrhein (1980), the primary effect of glyphosate on plants seems to be inhibition of 5-enolpyruvylshikimate-3-phosphate synthase (EPSPS), an enzyme of the shikimate pathway for biosynthesis of aromatic amino acids. EPSPS catalyzes the transfer of the enolpyruvyl moiety of phosphoenolpyruvate (PEP) to the 5-hydroxyl of shikimate 3-phosphate (S3P) to produce 5-enolpyruvylshikimate3-phosphate (EPSP) and inorganic phosphate (Schönbrunn et al. 2001). The enzyme is present in plants and microorganisms, but not in human or animal cells (Cole 1985). This mode of action is supported by the fact that most genetically modified GR crops carry a glyphosate-insensitive EPSPS derived from *Agrobacterium* spp. (Funke et al. 2006). Disruption of the shikimic acid pathway causes accumulation of shikimic acid and its derivatives and inhibits the biosynthesis of chorismic acid, precursor of the aromatic amino acids phenylalanine, tryptophan, and tyrosine. The resulting deficit of aromatic amino acids slows protein synthesis and can bring it finally to a halt, leading ultimately to the plant’s death. As chorismic acid also feeds into secondary phenolic pathways, the synthesis of various phenolic compounds and other secondary metabolites from aromatic amino acids is also disrupted (Hoagland and Duke 1982; Cole 1985; Komives and Schröder 2016). This can lead to a lack of defense molecules (e.g., phytoalexins), lignin derivatives, and plant hormones such as salicylic acid (SA) that functions as signal molecule (Dempsey et al. 2011) and thus compromises pathogen defense of treated plants (Johal and Huber 2009). The plant hormone auxin (indole-3-acetic acid, IAA), most likely derived from tryptophan (Mano and Nemoto 2012; Zhao 2012), may also be affected.

Glyphosate competitively inhibits the EPSPS, because it binds more tightly to the enzyme than one of its substrates, phosphoenolpyruvate (PEP), thereby inhibiting the enzyme’s catalysis (Schönbrunn et al. 2001). This specific inhibition, presumed to be the reason for the suitability and specificity of glyphosate as a herbicide, is based on the structural similarity of glyphosate with PEP, although glyphosate may not be regarded a mere analog of PEP, but rather appears to mimic an intermediate state of PEP (Pollegioni et al. 2011). Steinrücken and Amrhein (1984) tested seven enzymes from one or two organisms for inhibition by glyphosate, leading them to conclude that among PEP-utilizing enzymes EPSP synthase’s sensitivity towards glyphosate is unique. Three enzymes in *Escherichia coli*, including the DHAP synthase, required glyphosate concentrations far in excess of 1 mM, indicating that inhibition was related to the substance’s ion-chelating property (Steinrücken and Amrhein 1984). The DAHP synthase catalyzes the condensation of PEP and erythrose-4-phosphate (E4P) to 3-deoxy-d-arabino-heptulosonate 7-phosphate (DAHP), which is the initial reaction of the aromatic amino acid biosynthetic pathway. However, two DAHP synthase isozymes from other organisms were inhibited at 1 mM glyphosate and explained by competitive substrate inhibition: one in mung bean in competition with E4P (Rubin et al. (1982) and the other one in the yeast *Candida maltosa* in competition with PEP (Bode et al. 1984). Both organisms have insensitive DAHP synthase isoenzymes as well. Later reviews ignore that the two DAHP-synthases are inhibited by glyphosate, but merely state that apart from EPSPS, no other PEP-depending enzyme is inhibited by glyphosate (Songstad 2010 and Dyer 1994, cited therein) without referring to any particular study. Pollegioni et al. (2011) modify this statement by saying that apart from EPSPS, no other enzyme is inhibited by glyphosate *to a considerable extent*. However, inhibition of the DAHP synthases in mung bean and *C. maltosa* at 1 mM glyphosate is 85 and 21%, respectively, which is considerable. Taken together, it seems that no further PEP-utilizing enzymes or enzymes from other organisms were tested for inhibition by glyphosate since the work of Steinrücken and Amrhein (1984). This is surprising given that two DAHP synthase isozymes were found to be sensitive to glyphosate and that PEP is the substrate of a number of enzymes.^12^ The question whether, apart from the claimed specificity of glyphosate for EPSPS, other PEP-depending enzymes might be sensitive too is not dealt with in the Renewal Assessment Report (EFSA 2015b).

There are, however, reports indicating that adverse effects of GBH on plants may not be sufficiently explained by the inhibition of EPSPS and interference of the shikimic acid pathway but may also be linked to the presence of soil microbes (Schafer et al. 2012) and be connected to other properties of glyphosate, such as its chelating potential.

### Impacts on non-target organisms

It is widely known that main impacts of GBH use on biodiversity and, in particular, on invertebrate and vertebrate animals are indirect and strongly linked to the loss of wild plants that provide food and shelter to them (e.g., Schütte et al. 2017). However, direct impacts of herbicides seem also possible and various modes of action, affecting non-target organisms (i.e., humans, vertebrate and invertebrate animals, microorganisms) have been reported. Glyphosate and GBH induce oxidative damage in rat liver and kidneys by disrupting mitochondrial metabolism (Peixoto 2005; Olorunsogo 1990; Olorunsogo et al. 1979). Moreover, they disrupt endocrine-signaling systems in vitro, including multiple steroid hormones, which play vital roles in the biology of vertebrates (e.g., Romano et al. 2012; Thongprakaisang et al. 2013; Gasnier et al. 2009; Walsh et al. 2000). Also, non-mammalian animals (i.e., amphibians, fish) were found to be affected by GBH via endocrine disruption (Soso et al. 2007; Navarro-Martin et al. 2014). The glyphosate of GBH is suspected to impact microorganisms, both in terrestrial ecosystems (Newman et al. 2016a) and in the gastrointestinal microbiome in vertebrates (Shehata et al. 2013; Krüger et al. 2013b; Ackermann et al. 2015), probably because the shikimic acid pathway is present in microorganisms as well. After all, glyphosate resistance in many GMOs is based on the expression of an insensitive EPSPS from *Agrobacterium tumefaciens*.

### Glyphosate as chelating agent

In their patent application for aminomethylenephosphinic acids (including glyphosate), Toy and Uhing (1964) claimed “the new compounds have a wide variety of uses such as chelating agents, wetting agents, biologically active compounds and as chemical intermediates for the production of aminomethylenephosphinic acids and derivatives thereof.” At that time, the herbicidal activity of glyphosate was not yet recognized.

### Chelating agents affect bioavailability of elements in soils

Many micronutrients, but also toxic metals, are tightly bound to the soil matrix (organo-mineral particles), thus being not accessible for uptake into plants and other soil organisms. Chelating agents (e.g., EDTA, citric acid, humic substances) are able to compete for binding sites in the soil, hence, remobilizing the particle-bound elements into the soil solution and enhancing their solubility. This property is beneficially used for the purpose of phytoremediation of metal-polluted soils (Evangelou et al. 2007). But the presence of chelating agents in soil might also have deleterious effects by remobilizing toxic metals for uptake into soil organisms (Oviedo and Rodriguez 2003). However, although chelation can increase metal solubility in soils, at the same time plant uptake of chelated metals is usually inhibited and can only be increased when complex splitting in the rhizosphere is possible (Neumann and Römheld 2007). Whether glyphosate, with its chelating properties, is able to impact metal bioavailability in soils and thus indirectly contribute either to higher toxicity or nutrient limitation for soil organisms and plants is unclear but requires further attention in future studies.

Studies with earthworms suggest that the toxicity of copper in soil was rather alleviated in the presence of glyphosate (Zhou et al. 2013). This fits with the observation that inhibition of metal uptake by complexation with organic chelators released from plant roots and soil microorganisms also represents the basis for detoxification of toxic metals, such as Al^3+^ (Ma 2000). In aquatic ecosystems, where both heavy metals and glyphosate can co-occur, glyphosate and Roundup can control both the toxicity and the bioavailability of heavy metals, e.g., Cd, Cu, Cr, Ni, Pb, Se, and Zn (Tsui et al. 2005). Mixtures of glyphosate and the metalloid arsenic (As) showed synergistic toxicity to the nematode *Caenorhabditis elegans* (Wang et al. 2017).

Whether glyphosate has the potential to impact the bioavailability of certain metals in soils depends on its concentration and binding strength in relation to naturally occurring chelating agents. Below, the issue is described and available data are compared.

Many chelating agents can be found in soils, functioning, among others, as solubilizers for hardly accessible nutrients in soils (mainly P and Fe) or detoxifiers of toxic elements, such as Al. They are secreted into the soil by plants, bacteria, and fungi (e.g., Jones and Darrah 1994; Neilands 1995; Cesco et al. 2012) or introduced by industry for metal leaching (e.g., Lundström 1993) and influence the mineral partitioning between soil particles and solution. Roots release organic acid anions in response to a number of well-defined environmental stresses (e.g., Al, P, and Fe stress, anoxia) using multiple mechanisms; the responses are highly stress and plant species specific (Jones 1998). For Fe acquisition, plants evolved different mechanisms, including (i) the release of non-specific chelators such as organic acid anions and phenolics in combination with rhizosphere acidification and increased reductive capacity at the root surface, (ii) the release of more specific iron chelators, such as phytosiderophores, and (iii) the symbiosis with microorganisms which are highly efficient in Fe acquisition (e.g., by siderophores) (Römheld 1987).

The presence of amino, hydroxy-, di-, and tricarboxylic acids, sugars, and phenolic acids in root exudates of plants is known for a long time (e.g., Vancura and Hovadik 1965; Smith 1976). It is known that various adapted plant species and cultivars are able to increase the release of carboxylates with metal-chelating properties, such as citrate, oxalate, malate, or malonate in case of nutrient deficiency in sufficient amounts to increase nutrient availability in the rhizosphere (Lipton et al. 1987; Neumann and Römheld 2007). These organic acid anions can change chemical processes in soils by complexing metal ions (Stumm 1986; Martell et al. 1988) and by remobilizing particle-bound ions, although to a much lower extent than synthetic chelators, such as EDTA (Table 1). Highest release rates of citrate were found in white lupin and members of the *Proteaceae* in response to P limitation, representing plant species with a proven potential for mobilization of Fe-, Al-, and Ca-phosphates in soils (Dinkelaker et al. 1989; Roelofs et al. 2001). Soil solution concentrations of metal-chelating carboxylates in the rhizosphere of cluster roots reached millimolar concentrations equivalent to soil accumulation of 50 to 90 mmol/kg soil (Dinkelaker et al. 1989, 1997; Gerke et al. 1994; Li et al. 1997a, 1997b). However, in most soils, even total carboxylate concentrations hardly exceed the micromolar range (Jones et al. 2005). In forest soils, oxalic and formic acid showed the highest concentrations, with 5 to 105 and 95 to 295 μmol/kg soil, respectively, depending on the soil horizon that was analyzed (Fox and Comerford 1990). Free amino acids were detected in soil solutions in concentrations ranging from 0.2 to 7.3 μmol/L (e.g., glycine 3.1 μmol/L; total amino acid concentration 38 μmol/L; Jones et al. 2005).

**Table 1:** Reported stability constants for various chelating agents and metals

Metal	EDTA^a,1^	Citric Acid^a,1^	Oxalic Acid^b,3^	Formic Acid^c,2^	Glycine^d,3^	Siderophores^e,1,2^	Glyphosate^2^	AMPA^g,2^
Ca^2+^	12.4	4.9		0.3	1.4	6.0	3.3^f^	1.6
Cd^2+^	18.2	5.0	3.0	1.2	7.1–8.1	7.9	7.3^a^	5.1
Co^2+^	18.2	6.3	4.7	0.7	8.4–9.3		7.2^a^	4.6
Cu^2+^	20.5	10.9	6.3	1.4	15.2–15.6	14.1–22.3	11.9^f^	8.1
Fe^2+^	16	6.1	> 4.7		4.3^a^	7.2–8.3	6.9^a^	
Fe^3+^	27.7	13.2	9.4		10.9^a^	23–52		
Mg^2+^	10.6	4.9	2.6	0.3	3.4–4.0		3.3^f^	1.9
Mn^2+^	15.6	5.0	3.9		3.7^a^	17.3–47.5	5.5^f^	3.6
Ni^2+^	20.1	6.6	5.2	0.7	10.6–11.2		8.1^a^	5.3
Zn^2+^	18.2	6.1	4.9	0.7	8.9–10.0	4.4–19.8	8.4^f^	4.9

Stability constants were measured by potentiometric pH titration at a ionic strength of ^1^ *I* = 0; ^2^*I* = 0.1 M KNO_3_; ^3^information not given

^a^From Duke et al. (2012)

^b^From http://www.coldcure.com/html/stability_constants.html#what

^c^From Bunting and Thong 1970

^d^From Perkins 1952

^e^Values taken from several sources: Chen et al. (1994), Hernlem et al. (1996)^2^, Shenker et al. (1996)^2^, Kraemer (2004)^1^, Parker et al. (2004)^1^

^f^Taken from Madsen et al. (1978)

^g^From Song et al. (1994)

A much higher potential in increasing iron acquisition is provided by siderophores. Siderophores (from the Greek: “iron carriers”) are defined as relatively low molecular weight, mainly ferric ion-specific chelating agents secreted by bacteria and fungi growing under low iron stress (Neilands 1995; Kraemer 2004). The role of these compounds is to scavenge iron from the environment and to make the mineral, having a limited solubility in most soils with a pH > 5.5 and being almost always essential, available to the microbial cell (Winkelmann et al. 1987). However, they also form complexes with other essential elements (i.e., Mo, Mn, Co, and Ni) (Bellenger et al. 2008; Braud et al. 2009a, 2009b). It could be shown that plants can also benefit from bacterial siderophores in terms of an improved Fe supply (Rroco et al. 2003). Although increasing evidence suggests a considerable contribution of soil microorganisms to Fe acquisition in plants, a direct uptake of Fe siderophores by plants in significant amounts still remains speculative (Jin et al. 2014) since most plant species rely on uptake of free Fe^2+^ after root-induced complex splitting and/or microbial degradation of the siderophores (Hördt et al. 2000; Neumann and Römheld 2007). Therefore, the plant availability of Fe bound by siderophores strongly depends on the plant’s capacity for reductive complex splitting in the rhizosphere, which is determined by its genotype, the complex stability, soil pH, buffering capacity, and redox potential. Accordingly, detrimental effects by microbial siderophore producers on plant Fe acquisition have been repeatedly reported too (Becker et al. 1985; Walter et al. 1994; Windisch et al. 2017) and may be attributed to competitive interactions between plants and microorganisms.

Siderophores can be divided into three main families depending on their characteristic functional group, i.e., hydroxamates, catecholates, and carboxylates (Ahmed and Holmström 2014), with more than 500 different types of siderophores being known and 270 being structurally characterized (Ahmed and Holmström 2014). Siderophores selectively acquire and mediate iron transport and deposition inside the cell (Winkelmann et al. 1987). In order to efficiently capture Fe chelated by other ligands, siderophores must have very high affinities for binding. Indeed, hydroxamates and catecholates show stability constants with Fe(III) of 10^30^ and 10^40^, respectively, which is comparable to constants of EDTA and EDDHA (Robert and Chenu 1992). Although stability constants for other metals are lower (Neubauer et al. 2000), siderophores still might influence bioavailability of these metals for soil organisms. Studies on a number of bacteria and fungi show that metals other than iron can stimulate siderophore production (e.g., Huyer and Page 1988).

Also graminaceous plant species are able to release a specific group of siderophores (mugineic acids) with similar properties, termed phytosiderophores (PS) (Neumann and Römheld 2007). They are released particularly under conditions of Fe and Zn deficiency and exhibit high stability constants for Fe complexes at high soil pH levels, strongly limiting the solubility of Fe and other micronutrients. Competitive interactions between microbial siderophores and phytosiderophores for metal complexation have been reported by Yehuda et al. (1996) and Fe competition between siderophores and ferritin, lactoferrin, hemoglobin, and other Fe storage proteins also plays a major role in the interaction between bacterial pathogens and their vertebrate hosts (Skaar 2010). Metal-PS complexes represent the only known example within the plant kingdom, where chelated metals rather than free metal ions are taken up by plant roots; it is mediated by a specific metal-PS transporter (YS1) (Curie et al. 2001). Siderophore concentrations in soils are expected to range from 3 to 700 mg/kg soil (approx. 3–700 μmol/kg), if assuming concentrations of 4 to 875 mg/g bacterial biomass (Bossier et al. 1988 as cited in Hersman et al. 1995) and a mean bacterial soil content of 0.8 g/kg (Stevenson 1986 as cited in Hersman et al. 1995). Powell et al. (1980), however, found 0.18 μmol/kg soil of hydroxamate siderophores in aqueous soil extracts still sufficient to affect plant nutrition.

Apart from the adaptive release of metal chelators from plant roots and soil microorganisms, a major fraction of metal-chelating compounds in soils is provided by humic substances as intermediate products of organic carbon turnover in soils, which can represent up to 80% of total soil organic matter (Steelink 2002).

Thus, soil life itself provides many sources for chelating agents, leading to measurable concentrations in soils and soil solutions. To evaluate a potential glyphosate-mediated alteration of metal bioavailability in soils it is essential to compare stability constants of metal complexes (Table 1) and residual concentrations of glyphosate and AMPA with those of natural chelators in soils. In countries where GR crops are grown, high soil concentrations of glyphosate have been recorded, i.e., up to 1.5 mg/kg (9 μmol/kg) glyphosate and 2.25 mg/kg (20 μmol/kg) AMPA in Argentina (Aparicio et al. 2013). In Europe, top soil levels varied strongly between countries and main crops grown and reached up to 2.05 mg/kg glyphosate and 1.92 mg/kg AMPA in vineyards in Portugal (Silva et al. 2017). Shortly after glyphosate application or on soils with long-term intensive glyphosate use, even higher levels have been found (Peruzzo et al. 2008; Afzal 2017). For Finnish soils, Laitinen et al. (2009) recorded 2 μmol/kg glyphosate in the 0 to 5 cm top soil 240 days after application. Similar levels of glyphosate residues have been reported in an earlier review on various field soils (Franz et al. 1997) ranging from 4.7 to 14.1 μmol/kg after 15 days and 0.3 to 6.5 μmol/kg 3 to 7 months after the last application. Less data are available on glyphosate (0.001 to 0.02 μmol) and AMPA (0.004 to 0.014 μmol) in soil solutions, which have been collected with lysimeters (Bergström et al. 2011) or by water extraction (Afzal 2017).

Another interesting aspect in this context is to compare residual levels in the soil of glyphosate and AMPA as potential chelators with ranges of the plant-available fractions of potential counterions for glyphosate and AMPA; a range of published values is summarized in Table 2. The comparisons show that the stability constants of various metal complexes with glyphosate and AMPA or potent natural metal chelators in soils are in a comparable range (Table 1). They also show that concentrations of glyphosate and AMPA in the soil (overall range is 0 to 40 μmol/kg (Table 2)) fall in the lower range of expected siderophore concentrations ranging between 3 and 700 μmol/kg (see above). However, concentrations of plant-available counterions are, except for Zn, several orders of magnitude higher than the reported residual soil concentrations of glyphosate and AMPA, even at high contamination levels (Table 2). The available data implicates that except for Zn a significant direct long-term impact of glyphosate and AMPA on bioavailability of metals in soils seems to be rather unlikely and the reported negative side effects on non-target organisms need to be explained by another mechanism. However, most data do not consider glyphosate’s tendency to accumulate and persist in microhabitats such as the root zone of plants exposed to glyphosate (Kremer 2017). Therefore, more sophisticated data might modify this implication.

**Table 2:** Reported levels of residual top soil concentrations of glyphosate and AMPA and the respective plant-available fractions of counterions in soils (data compiled from various references: Franz et al. 1997 (16 soils), Aparicio et al. 2013 (32 soils), Peruzzo et al. 2008, Afzal 2017 (13 soils), Laitinen et al. 2009 (different soil depths)

Component	Soil concentration (μmol/kg dry weight)
Glyphosate	
High contamination level	10–30
Low contamination level	0–5
Glyphosate + AMPA	
High contamination level	20–40
Low contamination level	0.5–7
Plant-available counterions	
Zn	40–80
Mn	500–2000
Fe	900–1800
Mg	4000–150,000
Ca	25,000–250,000

### Chelating properties of glyphosate in plants

Due to its three chemical groups (amine, carboxylate, and phosphonate), glyphosate can strongly bind to cations, such as Ca, Fe, Zn, Al, and Mn. Although, compared to EDTA (ethylenediaminetetraacetate), glyphosate has been described as a weak chelator (Duke et al. 2012), it may nevertheless bind important micronutrients (and potentially also macronutrients), thus impeding, for instance, their availability in glyphosate-treated sensitive plants (Eker et al. 2006; Tesfamariam et al. 2009; Cakmak et al. 2009). In commercial products, the organic acid glyphosate is often used in its salt form, with isopropylamine (IPA, synonym isopropylammonium) as a cation, but IPA is also a chelator, e.g., of iron, and may thus increase the chelating potential of glyphosate (Sonier and Weger 2010). As micronutrients are important co-factors of plant enzymes and play a role in stabilizing proteins and other physiological functions (Table 3), an undersupply with nutrients such as Mn, Fe, Cu, and Zn could impact photosynthesis and plant growth and potentially increase plant disease (Johal and Huber 2009). According to Duke et al. (2012), however, glyphosate use does not increase plant disease in GR crops. Whether food and feed derived from glyphosate-treated plants (that may still contain glyphosate residues) could also lead to a reduced micronutrient uptake by humans and animals has not been elucidated.

**Table 3:** Main functions of micronutrients in plants

Element	Symbol	Main physiological functions in plants	References
Boron	B	Cell wall synthesis and structure, cell membrane function, lignification, IAA formation, nodule development, other (secondary) processes	1, 2, 3, 4
Cobalt	Co	Nodule initiation	3
Copper	Cu	Essential for photosynthesis, mitochondrial respiration, C and N metabolism, oxidative stress protection, catalytic metal in many oxidases, pollen fertility, plant defense, synthesis of phenolics, photosynthesis	1, 2, 4, 5
Iron	Fe	Central part of hemoproteins (e.g., cytochromes), involved in photosynthesis, mitochondrial respiration, N assimilation, hormone biosynthesis, osmoprotection, pathogen defense, oxidative stress protection	1, 2, 4, 6, 7
Manganese	Mn	Cofactor/activating role for at least 36 enzymes, protection from free radicals, involved in shikimic acid pathway and production of phenolics, fatty acid synthesis, N metabolism, C fixation, chloroplast function	1, 2, 4, 6, 7
Molybdenum	Mo	N assimilation and fixation (e.g., nitrogenase, nitroreductase), biosynthesis of abscisic acid	1, 2, 4
Nickel	Ni	Urease activity, hydrogenase activity in legume nodules	2, 4
Zinc	Zn	Component of many proteins involved in DNA replication, transcription, translation, C fixation, carbohydrate and protein metabolism, oxidative stress protection, disease resistance	1, 2, 4, 6, 7

References: (1) Rice (2007), (2) Hänsch and Mendel (2009), (3) O’hara et al. (1988), (4) Broadley et al. 2012, (5) Evans et al. 2007, (6) Solymosi and Bertrand 2012, (7) Elmer and Datnoff 2014

Since discovering the herbicidal activity of glyphosate in the 1970s (John 1974), various studies and reviews (e.g., Duke et al. 2012; Gomes et al. 2014; Reddy and Duke 2015) appeared analyzing potential impacts of glyphosate and GBH on uptake, root-to-shoot transport, and levels of micronutrients in treated plants, be they GR crops or non-genetically modified (non-GM) glyphosate-sensitive plants. Study results depend, among others, on the total amounts of glyphosate applied and whether glyphosate has been applied once or several times during the growing season, since a dose applied once and at an earlier plant stage may lead to different effects than the same dose split in two applications with the second one several weeks later. Impacts on nutrient accumulation may vary with the Roundup formulations used (Cavalieri et al. 2012). However, due to limitation of the root system in potted plants to the size of the pot, results of such studies may not be simply extrapolated to field conditions (Reddy and Duke 2015).

### Greenhouse and field studies with glyphosate-resistant plants

Greenhouse studies showed that glyphosate application in the recommended dosage can exert unexpected negative effects on glyphosate-resistant (GR) crops. Glyphosate is applied at different rates and in different formulations. When provided in the cited literature, the used formulation is specified here. Varying with culture systems and different soils (calcareous Luvisol and acidic Arenosol), glyphosate applied as Roundup Ultramax inhibited root biomass, root elongation, and lateral root formation in GR soybean, associated with decline of Mn concentrations in young shoots of plants grown in hydroponics and of Zn in plants grown in soil culture (Bott et al. 2008), although tissue concentrations did not drop below critical levels. In hydroponic culture experiments, negative effects were prominent under conditions of sufficient Mn supply (0.5 μM), but not at low Mn supply (0.1 μM), which may indicate that the enzymatic conversion of glyphosate to phytotoxic AMPA in soybean was possible under sufficient Mn supply, but not at low Mn supply, perhaps due to Mn demand of the enzymes converting glyphosate to AMPA.

Recently, a series of Brazilian greenhouse studies on effects of glyphosate (commercially formulated isopropylamine salt) on various GR soybean lines has been published. Zobiole et al. (2010a) reported that GR soybeans of various cultivars grown on different soil types experienced “yellow flash” and significant decreases in photosynthetic parameters (chlorophyll, photosynthetic rate), Ni content, and nodule numbers with glyphosate treatment (1.2 kg a.e./ha in a single or sequential application). Shoot, root, and nodule dry weight were also lower, the latter potentially linked to lower Ni availability to symbiotic microorganisms that require Ni for efficient N fixation (Lavres et al. 2016). Other studies showed that treatment with increasing glyphosate rates (five doses from 0.6 to 2.4 kg a.e./ha, applied either in a single dose or sequentially) decreased photosynthetic parameters in leaf tissues of GR first-generation soybean in a linear fashion and reduced accumulation of macronutrients (in the order Ca > Mg > N > S > K > P) and micronutrients (in the order Fe > Mn > Co > Zn > Cu > B > Mo), compared to the near-isogenic non-resistant parental line (Zobiole et al. 2010b). Water use efficiency was reduced too (Zobiole et al. 2010c). Impacts of single versus sequential application varied, with single applications of the full dose leading to stronger effects on macro- and micronutrient accumulation. Glyphosate treatment can also reduce macro- and micronutrient concentrations in seed of GR soybean, although the pattern was not as clear as in leaf, shoot, and root dry biomass (Zobiole et al. 2010d). Glyphosate impacts on mineral nutrition and total N, Mn, Cu, Zn, and Fe contents in GR soybean have been observed by other groups, too (Serra et al. 2011).

Additional greenhouse studies with three doses of glyphosate (0.8 to 2.4 kg a.e./ha, Roundup WeatherMax or non-specified commercial formulation) applied to GR soybean revealed that second-generation (RR2) soybean, developed and promoted for higher yields (Zobiole et al. 2012), reacted no better compared to first-generation (RR1) GR soybeans with regard to impacts on chloroplasts, photosynthesis (Zobiole et al. 2010e) and decreases in macro- and micronutrient accumulation, nodulation, and shoot and root biomass (Zobiole et al. 2011). Glyphosate isopropylamine salt impacts on shoot macro- and micronutrient levels, photosynthetic parameters, and biomass production of GR soybean varied somewhat between different cultivars (the early maturity group was more affected) and with soil type (Zobiole et al. 2010a, f). In GR-RR1 soybean, lignin content in root and stem tissues and amino acid production in leaves were reduced by more than half, if glyphosate rates increased to commercial levels of 1.35 and 1.8 kg a.e./ha applied in a single dose (Zobiole et al. 2010g). With high glyphosate rates, macro- and micronutrient accumulation in leaves was significantly decreased in both RR1 and RR2 cultivars (Zobiole et al. 2011). The negative impacts on photosynthesis, e.g., reduced photosynthetic rate, stomatal conductance, and transpiration rate, observed very often after glyphosate application, may be linked to direct damage to chloroplasts by glyphosate or in case of GR soybean more likely to AMPA toxicity, since soybean shows an exceptional ability for partial degradation of glyphosate into its phytotoxic metabolite AMPA (Reddy et al. 2004). In GR soybean, treated with glyphosate plus 0.5% Tween 20, both glyphosate and AMPA can accumulate to millimolar concentrations in young leaves (Reddy et al. 2004). For this reason, the observed symptoms may also be a consequence of immobilization of essential nutrients by metal chelation, since Mg, Mn, Fe, and Zn and other minerals are required for chlorophyll production and function, light reactions, and C assimilation (Broadley et al. 2012; Solymosi and Bertrand 2012).

Impacts on micronutrient accumulation were also observed in the field. RR2-GR soybean, treated at different growth stages with doses ranging from 0.8 to 2.4 kg a.e./ha (the latter one to represent the “worst-case scenario”), showed that photosynthetic rate, nodulation, macronutrient (N, P, K, Ca, Mg, S) and micronutrient (Zn, Mg, Fe, Co, B) accumulation in plant tissue decreased with higher doses and treatment at later growth stages, although concentrations were within the nutrient sufficiency ranges for soybean (Zobiole et al. 2012). Recently, Duke et al. (2017) reported that, in a 2-year field study, they did not find consistent effects of glyphosate, the glyphosate-resistance transgene, or field crop history on measured mineral content of leaves or harvested seed of GR soybean. Applying in total 1.74 kg a.e./ha per season, effects on minerals were reported to be small and inconsistent between years, treatments, and mineral and were thought to be random false positives.

In GR maize, stacked with insect resistance traits (NK603 stacked with MON810 and/or Mon863), a single glyphosate (Roundup Ultramax) application did not change mineral content in maize grain (Ridley et al. 2011). Under agronomic conditions, however, multiple applications are very common (Benbrook 2016). In a 15-year field study with continuous GR corn and soybean, mimicking a typical US crop production system, herbicide management affected available P, Fe, K, NO_3_-N, and SO_4_-S concentrations, but had no significant effect on soil pH, organic matter, and exchangeable Ca, Mg, Mn, or Zn (Obour et al. 2016). pH and soil mineral concentrations were affected by crop rotation and soil sampling depths. In the glyphosate-only treatments (other treatments included non-glyphosate herbicides and glyphosate alternated every other year with non-glyphosate herbicides, but there was no true control with herbicide-free plots), P and Fe concentrations increased in the top soil (7.5 cm). Mineral uptake by crop plants was not determined.

Although Duke et al. (2012) found conflicting literature regarding glyphosate effects on mineral nutrition, rhizosphere biota, and plant disease in GR crops, they concluded that data collected with GR crops (mainly Roundup Ready soybean lines) indicate that glyphosate application does not restrict the availability of micronutrients.

### Studies with non-resistant plants

There are only few studies on the potential impact of glyphosate-based herbicides on the mineral content of non-target plants, such as non-resistant crops or ornamental plants, although they can be affected by spray drift (Thomas et al. 2005) and by soil residues (Tesfamariam et al. 2009). Plant to plant transfer of glyphosate via root exudation has been observed too, exerting inhibitory effects on the shikimate pathway, uptake of micronutrients (Mn), and growth of non-target sunflowers (Neumann et al. 2006). Effects were prominent in acidic sandy soils, whereas in calcareous soils, high Ca levels may have prevented glyphosate uptake by non-target plants, due to rapid precipitation of glyphosate released from target plant roots. Simulated spray drift on non-resistant soybeans (0.6% of the recommended application concentration) caused significant reductions of dry weight, chlorophyll contents, and concentrations of some macronutrients (Ca, Mg) and micronutrients (Mn, Fe) in young leaves (Cakmak et al. 2009). At 1.2% of the recommended application concentration, seed concentrations of Mg, Ca, Mn, and Fe were reduced by 12.5 to 49%, whereas seed concentrations of N, K, Zn, and Cu were increased. Shoot and seed yield were about 3-fold and 5-fold lower, respectively. Spray volumes, however, were high, leading to an almost full absolute dose of glyphosate application, even at low application concentrations, but the efficiency might have been affected by the corresponding dilution of the formulation.

In similar treatments of non-GM non-GR sunflowers, simulated spray drift led to significantly reduced growth and lower chlorophyll content in young leaves and sprout tips, the transportation of Fe and Mn from root to sprout was almost entirely inhibited within a single day (Eker et al. 2006). As a process of Fe acquisition, dicots and non-grass monocots use the reduction of Fe(III) to Fe(II) at the root surface which is mediated by ferric reductase induced under Fe deficiency. More soluble Fe(II) can then be transported across the plasma membrane. In Fe-deficient sunflower roots, drift rates of foliar-applied GBH inhibited ferric reductase activity, potentially linked to the formation of insoluble, stable Fe-complexes that are not available for reduction by ferric reductase (Ozturk et al. 2008). Treatment of iron-limited green alga *Chlorella kesslerii* with various ferric chelators, among them glyphosate, led to inhibition of plasma membrane ferric reductase, depending on the amount applied (Sonier and Weger 2010). There were also differences with regard to (pure) glyphosate acid and glyphosate-isopropylamine (IPA) salt, with glyphosate-IPA having a slightly larger effect, but IPA alone, being a chelator too, was also inhibitory of ferric reductase. The authors suggest that low concentrations of glyphosate likely solubilize Fe(III), making it available for plant growth, but that higher (yet sub-lethal) concentrations decrease iron acquisition by inhibiting ferric reductase activity. Impairments of Fe uptake and translocation in plants could be a major reason for the increasingly observed Fe deficiency chlorosis in cropping systems associated with widespread glyphosate use (Jolley et al. 2004). Impacts of glyphosate spray drift doses (72 g a.e./ha and 288 g/ha a.e.) on nutrient concentrations in shoots of different turfgrass species varied considerably, in some species glyphosate applied as Roundup Ultra reduced concentrations of nutrients, e.g., Ca, Mg, Mn, and Fe, significantly (Senem et al. 2009). For all studies cited above, it remains to be established to which extent the observed effects are a consequence of the well-documented phytotoxic mode of action of glyphosate as inhibitor of the EPSPS and/or of the toxic formulants, or if metal-chelating properties are involved at least partially.

Treating weeds with glyphosate as Roundup Ultramax shortly before seeding the crop can impair seedling growth, as shown by Tesfamariam et al. (2009) who found up to 90% reduction in root and shoot biomass of sunflower (*Helianthus annuus*). Detrimental effects were more pronounced after glyphosate application to weeds, compared to direct soil application, indicating that the root tissue of glyphosate-treated weeds could represent a storage pool for the herbicide, also reported by Doublet et al. (2009) with Roundup Biovert 360. Similar effects have been reported by Bott (2010) for short waiting times after pre-crop application of Roundup UltraMax at application rates of 2 and 4 L/ha in winter wheat, both in pot experiments and under field conditions, particularly expressed at high weed densities. Although in pot experiments a rhizosphere transfer of glyphosate from the decaying roots of target weeds to the roots of non-target plants is particularly evident due to intense root intermingling in the limited soil volume, similar conditions can also apply for field applications. Since the highest rooting densities usually occur in the top soil layers with the highest nutrient supply, intense root intermingling can be expected at high weed densities also in the field (Bott 2010) or in cases where glyphosate is applied for desiccation during grassland conversion for agricultural use. Moreover, Watt et al. (2006) reported that up to 50% of wheat roots in no-tillage systems in Australia occupied pre-crop root channels, another factor increasing the risk of root contact of non-target crops with glyphosate-treated weeds. As shown before (Neumann et al. 2006), glyphosate impacts on non-target plants depend also on the soil type, as Mn nutrition in non-GM sunflower was more strongly impaired in a soil with low buffering capacity and lower levels of available Mn (Arenosol) than in a well-buffered calcareous subsoil (Tesfamariam et al. 2009).

In general, if a soil is depleted in a given micronutrient to a content deficient for adequate plant growth, then GBH’s activity is likely to magnify the shortage. Although, under practice conditions, extreme micronutrient deficiencies in agricultural soils are usually balanced by fertilization, less obvious deficiencies may go unnoticed. For studies of GBH reactivity in soils, it would thus be important to have soil test values, but these data are usually lacking.

Sonier and Weger (2010) point out that most studies dealing with impacts of glyphosate have been performed with commercial glyphosate formulations that include not only the glyphosate acid but also the cation isopropylamine (IPA) that can act as a chelator too (and one or more formulants). Whether diverging results of studies dealing with the chelating activity of GBH could also be linked to which salt form of glyphosate (and which formulation) has been tested is unclear. Apart from the active ingredient glyphosate and possibly IPA, other additives of herbicide formulations are not known for being chelators but are rather described as being inert. This is, as far as we are aware, not regularly checked for the approval of plant protection products. It would add to our knowledge if the various additives in commercially used formulations are tested for their ability to enhance or reduce glyphosate’s chelating property in various matrices.

### Potential effects of glyphosate and GBH on plant–microorganism interactions

Glyphosate influences the soil microflora and their interactions with plants, suppressing some microorganisms and favoring others (Kremer and Means 2009; Yamada et al. 2009), perhaps also linked to varying sensitivities of microbial EPSPS enzymes to glyphosate (Clair et al. 2012; Nicolas et al. 2016). Glyphosate impacts nodulation and symbiotic nitrogen fixation, potentially due to the sensitivity of symbionts of both soybean and other legumes to glyphosate (Zablotowicz and Reddy 2004). Depending on glyphosate rates, reduced nitrogen fixation and/or assimilation in GR soybean systems has been reported (Zablotowicz and Reddy 2004; Means et al. 2007; Zobiole et al. 2012), in particular at above label use rates of glyphosate and under soil moisture stress (Zablotowicz and Reddy 2007). In non-GR soybean, nitrate assimilation and nitrogen fixation can be affected by a single application of a spray drift rate (0.1 kg a.i./ha) of glyphosate (Bellaloui et al. 2006). Although impacts of GBH on nitrogen fixation in soybean may be attributed to a glyphosate-sensitive EPSPS of certain strains of the symbiont *Bradyrhizobium japonicum* (Zablotowicz and Reddy 2007; Cerdeira and Duke 2006; Duke et al. 2012), chelating properties of glyphosate might contribute to the negative effects observed. It is well known that nodulation and nitrogen fixation are also dependent on various micronutrients, e.g., Fe, Cu, Co, Zn, Ni, and B (O’hara et al. 1988; Gonzáles-Guerrero et al. 2014).

Microorganisms other than symbionts of legumes, e.g., rhizosphere bacteria active in Mn transformation in soil, may also be affected by glyphosate, as observed by Zobiole et al. (2010h) in a greenhouse study with both RR1 and RR2 glyphosate-resistant soybean treated with Roundup WeatherMax. In a 10-year field study, beneficial fluorescent pseudomonads, associated with antagonism of fungal pathogens and manganese reduction to Mn^2+^ (taken up by plants), were significantly reduced, whereas bacteria oxidizing Mn^2+^ to Mn^4+^ (not taken up by plants) increased in the rhizosphere of glyphosate-resistant crops (Kremer and Means 2009). Mn is a critical co-factor and activates several enzymes in the shikimic acid pathway for the production of phenolics, cyanogenic glycosides, and lignin defense barriers (Elmer and Datnoff 2014). The requirement for Mn in various steps of lignin biosynthesis is reflected in lower lignin contents in Mn-deficient plants, particularly evident in their roots, which is an important factor responsible for the lower resistance of Mn-deficient plants to root-infecting pathogens (Broadley et al. 2012).

Pathogens such as the fungus *Gaeumannomyces graminis* (take-all disease of cereals) have been linked to the availability of Mn for plant uptake, since conditions that reduce Mn availability increase take-all (Johal and Huber 2009; Huber et al. 2012). Discussing reasons why, within the last years, an increase of take-all diseases of cereals following GBH applications has been observed, Johal and Huber (2009) suggest that it may be the result of (i) reduced resistance from induced Mn deficiency, (ii) inhibited root growth from glyphosate accumulation in root tips, (iii) modified virulence of the pathogen, or (iv) an increase in synergistic Mn-oxidizing organisms in the rhizosphere.

Other macro- and micronutrients, whose plant concentrations have been shown to be affected by GBH treatment, can also play a role in plant disease, e.g., Ca, Mg, Fe, Zn, and Cu (Elmer and Datnoff 2014; Huber et al. 2012). Mg, for instance, is an important contributor to overall plant health (Huber and Jones 2013). In a long-term rhizobox experiment with GR corn and soybean, the influence of GBH on gene expression of rhizosphere microorganisms was studied. Most transcripts from rhizosphere soil that differed in their abundance between the GBH-treated and the control plants were linked to carbohydrate, protein, and amino acid metabolism (Newman et al. 2016b). However, bacterial transcripts involving nutrients, including Fe, N, P, and K, were also affected by long-term GBH application. The authors discuss, whether, for instance, the downregulation of transcripts involved in Fe acquisition was linked to a reduced abundance or availability of Fe in the rhizosphere of GBH-treated crops, although upregulation of these transcripts might have been expected in order to acquire Fe in an environment with low amounts of available Fe. However, lower abundance of transcripts for Fe acquisition may also reflect a lower abundance of microorganisms with the capacity for siderophore production (e.g., Pseudomonades) as described above (e.g., Kremer and Means 2009).

In face of the various observations described above, finally the question remains whether the reported detrimental effects of GBH on the micronutrient status of non-target organisms or GR plants can be related with the metal-chelating properties of glyphosate. Reports on internal glyphosate concentrations after contamination of non-target plants are rare. But information on compartmentation and the concentrations of glyphosate in the young growing target tissues and the corresponding counterions would be a prerequisite to evaluate the risk of an internal nutrient immobilization by complexation with glyphosate. Wagner et al. (2003) demonstrated in uptake experiments with ^14^C-labeled glyphosate that plant growth depression was already induced after root uptake of 1 μg glyphosate per plant by maize seedlings at 14 days after sowing. Assuming a plant fresh biomass of 2 to 4 g at this growth stage, this would translate into a tissue concentration of approximately 15 to 30 μmol/kg plant dry matter and 80% of the accumulated glyphosate remained in the root tissue (Wagner et al. 2003). By contrast, Reddy et al. (2004) reported glyphosate concentrations of 700–1400 μmol/kg plant dry matter in young leaves of glyphosate-treated GR soybean 1–2 weeks after application.

The comparison of the glyphosate concentrations with the range of cationic counterions usually detected in the tissues of the youngest fully developed leaves in plants with sufficient nutrient supply, demonstrates an excess of 1 to 2 orders of magnitude for cationic micronutrients and 3 to 4 orders of magnitude for Ca and Mg at the low contamination level of maize (Table 4) described by Wagner et al. (2003). However, glyphosate concentrations in highly contaminated GR soybean plants (Reddy et al. 2004) were comparable with reported cationic micronutrient concentrations (Fe, Zn, Mn) in the leaf tissues (Table 4) and, due to limited phloem mobility, young leaves are particularly susceptible for micronutrient deficiencies. These findings suggest that internal micronutrient immobilization may be a realistic scenario in GR crops after application of regular glyphosate doses but not for low-level contamination of non-target crops by herbicide drift or rhizosphere transfer. Under these conditions, direct low-level toxicity effects of glyphosate and AMPA impairing root growth, nutrient uptake, and translocation processes are a more likely explanation. Nevertheless, testing these hypotheses would require more detailed comparative and localized measurements of both glyphosate/AMPA concentrations and the respective counterions, including speciation analysis in tissues and plant organs showing preferential glyphosate accumulation and, in addition, activity monitoring of metabolic pathways affected by limited micronutrient availability.

**Table 4:** Reported plant tissue concentrations of glyphosate and the related shoot concentrations of counterions (data calculated from various references: Wagner et al. 2003^1^ maize seedlings, Reddy et al. 2004^2^ GR soybean, young leaves 1–2 weeks after application Marschner 1995)^3^

Component	Tissue concentration (μmol/kg dry weight)
Glyphosate	
Drift contamination (maize)^1^	15–30
Target weeds, GR-soybeans^2^	700–1400
Counterions in plant tissue	
Zn^3^	300–1200
Mn^3^	450–3000
Fe^3^	700–4400
Mg^3^	80,000–240,000
Ca^3^	75,000–200,000

### Potential effects on animals and humans

Glyphosate (and AMPA) residues have been shown to be present both in GR crop plants and in non-resistant plants and their products, exceeding MRL values in some cases, in particular in GR soybean (Cuhra 2015; Bai and Ogbourne 2016) where residue levels of 0.4–8.8 mg/kg for glyphosate and 0.7–10 mg/kg for AMPA, resulting in up to 15 mg/kg total residues, have been reported (Bøhn et al. 2014). In certain cases total levels may even reach more than 50 mg/kg (Cuhra 2015). Thus, food and feed derived from GBH-treated plants can contain significant amounts of glyphosate and AMPA residues and probably also residues of formulants such as POEA (the latter are far from being routinely tested). If, in addition, food and feed derived from GBH-treated plants contain lower amounts of macro- and/or micronutrients, then their quality may be further compromised. However, only few data have been published with regard to micronutrient levels in GBH-treated plants, compared to conventional or organic crops. Testing individual Iowa soybean samples, Bøhn et al. (2014) found that glyphosate-resistant soybean samples had significantly lower Zn levels than organic probes, whereas differences in other micronutrients (Ba, Cu, Fe, Mn, Mo, Se) were relatively small.

Glyphosate residues have been found in urine of humans (Acquavella et al. 2004; Curwin et al. 2007; Mills et al. 2017). In lifestock and humans, lower amounts have been observed in urine of cows kept in a GMO-free region, compared to conventionally managed cows, and in urine of humans consuming predominantly organic food, compared to consumers of conventional food (Krüger et al. 2014). In Danish dairy cows, glyphosate amounts in urine were correlated with important blood serum parameters, with serum levels of Mn and Co being unexpectedly low, far below the minimum reference levels and too low for proper function and immune response, whereas serum levels of Se and Cu were within the reference range and Zn levels excessive in cows at some farms (Krüger et al. 2013a).

Jayasumana et al. (2014, 2015) report that simultaneous exposure to multiple heavy metals and GBH may contribute to chronic kidney disease that has been identified among paddy farmers in some parts of Sri Lanka where groundwater is found to be hard or very hard and contain Ca, Mg, Fe, and strontium (Sr) ions. Urinary excretion of heavy metals, e.g., cadmium (Cd), lead (Pb), and the metalloid As, and glyphosate was significantly higher in people living in the endemic areas, compared to those living in non-endemic areas. The disease was first observed in the mid-1990s several years after the introduction and large-scale application of agrochemicals, with GBH amounts exceeding the sum of all other leading pesticides imported to Sri Lanka in 2012. Jayasumana et al. (2014) propose a glyphosate-metal lattice to explain the possible (synergistic) role played by glyphosate, water hardness, and nephrotoxic metals (heavy metals may also be derived from fertilizers) in the pathogenicity of chronic kidney disease in Sri Lanka and potentially in other countries, such as Central America. It is known that, by remobilizing toxic metals for uptake into soil organisms, the presence of chelating agents in soil might have deleterious effects (Oviedo and Rodriguez 2003).

Metals such as Mg, Ca, Fe, Mn, Cu, Co, and Zn are also important nutrients for humans. In case human food would be derived from glyphosate-treated plants to a considerable extent, glyphosate residues and reduced levels of micronutrients could potentially also impact human health. In the USA, where a large proportion of food is likely derived from genetically modified crops, and in particular from glyphosate-resistant crops, several authors have asked the question whether there would be any link between the widespread use of GBH and the disease load in the US population (e.g., Samsel and Seneff 2013, 2015; Swanson et al. 2014). Samsel and Seneff (2015), for instance, mention a range of neuropathologies that are linked to Mn deficiencies and propose that the recently observed rate increases of such diseases in the USA could be due to a variety of environmental toxicants, with broadly used GBH potentially being one of them. In another paper, Samsel and Seneff (2013) discuss the potential role of glyphosate in celiac sprue and gluten intolerance, linked perhaps to Fe, Co, Mo, Se, and Zn deficiencies. Swanson et al. (2014) report to have used US government data on glyphosate application and on disease epidemiology and to have found correlations between the increased use of glyphosate and the increase in various diseases within the last years. However, correlations are no proof of a causative relationship. In addition, as glyphosate is not applied alone, but always as an ingredient of a complex formulated product, negative effects may also be due to the formulants whose composition and concentration are not generally known to the public. It is, however, well known that formulants, e.g., ethoxylated adjuvants such as POEA, can act as toxins exhibiting more than 1000 times higher toxicity to human cells than glyphosate (Mesnage et al. 2013). But measurements deal generally with glyphosate (and AMPA) only, and data about the formulants used are extremely rare. As GBH use most likely will not be abandoned any time soon globally and in view of strategies to develop plants which are resistant to higher glyphosate concentrations (Dun et al. 2014; Guo et al. 2015), it seems to be reasonable to investigate potential impacts on human and animal health further.

### Chelating properties of glyphosate in the reevaluation process

In the process of reevaluation of glyphosate, chelating properties of glyphosate have not been discussed widely. In its conclusions on the peer review of the risk assessment of glyphosate, EFSA (2015a) did not provide data on potential impacts of glyphosate on plant nutrient availability. The final addendum to the Renewal Assessment Report (EFSA 2015b, pp. 37, 256) contains a short section on the chelating properties of glyphosate: “Findings have shown that glyphosate can be transferred from the roots of target plants to the rhizosphere and non-target plants can also be influenced (e.g. reduced absorption of micronutrients – Mn and Fe deficiency). Glyphosate is a strong chelator to various divalent cations such as Ca, Fe, Cu and Mn. Glyphosate binds micronutrients in the soil and can cause micronutrient deficiencies in plants that increase their susceptibility to disease, especially on soils with pure nutrient content.” EFSA mentioned an additional point (EFSA 2015b, Volume 3, Annex B.3 p. 11): “It can reduce the plant’s production of lignin and phenolic compounds, which are also important for disease resistance.” EFSA’s conclusion for this issue reads: “However, the available scientific data suggest that the strong affinity of glyphosate and its metabolite AMPA to most soils prevents the uptake of these compounds by root systems of non-target plants.” This implies that, in the course of the renewal process of glyphosate, it would not be necessary to pay attention to the binding of micronutrients by glyphosate and potentially resulting micronutrient deficiencies. In total, on the 4322 pages of the final addendum (EFSA 2015b), the terms “chelator” and “chelating agents” appear less than five times each, in most cases referring to paper abstracts, whereas in the assessment sections “chelator” is mentioned only twice (pp. 37 and 256). In the ECHA opinion on glyphosate the terms “chelating agents” and “chelator” have not been mentioned at all (ECHA 2017). With respect to the applied formulations, Commission Regulation (EU) No 284/2013^13^ sets out the data requirements for the approval of plant protection products. This Regulation does not specify the necessity to test at a chemical level whether the chelating property of an active ingredient is modified in commercially used formulations.

Similarly, in the scientific opinions written by EFSA in the course of applications for cultivation of glyphosate-resistant GM crops, such as the glyphosate-resistant (GR) maize lines NK603 (EFSA 2009) and GA21 (EFSA 2011), and the GR soybean 40-3-2 (EFSA 2012), the chelating property of glyphosate as a potential second mechanism of action in addition to EPSPS inhibition has not been discussed. EFSA referred to potential effects of glyphosate, used on these GM crops, on soil microbial communities, such as rhizosphere-inhabiting bacteria and fungi, including those capable to live in mycorrhizal relationship and nitrogen-fixing rhizobia, but did not discuss in detail potential impacts of glyphosate being a chelator. The assessment of soybean 40-3-2, EFSA (2012, p. 45) mentioned the papers of Cakmak et al. (2009), Johal and Huber (2009), and Kremer and Means (2009), indicating that glyphosate can interfere with root enzymes involved in mineral uptake from the soil, immobilize nutrients, or may transform nutrients to plant unavailable forms by stimulating certain microorganisms. EFSA (2012) cited additional papers such as the ones of Gordon (2007), Zobiole et al. (2010f, 2011), and Bott et al. (2008, 2011) who reported that GBH applied to plant foliage could induce directly or indirectly deficiencies in micro- and macronutrients, e.g., manganese deficiencies by decreasing Mn-reducing rhizobacteria, requiring altered fertilizer applications. The question whether glyphosate residues in food and feed derived from these glyphosate-resistant GM crops may impact human and animal health has not been answered by EFSA.

## Conclusions

The review of available data indicates that, in addition to the competitive inhibition of the enzyme 5-enolpyruvylshikimate-3-phosphate synthase (EPSPS), leading to interference with the shikimic acid pathway in plants (and many microorganisms) and known as the main mode of action of the declared active ingredient glyphosate, other properties of this herbicide may be important too. Glyphosate is a chelating agent and as such binds macro- and micronutrients and can impact their uptake and availability in plants treated with glyphosate-based herbicides, be they genetically modified to resist the application of glyphosate or not. In particular, availability of micronutrients such as iron, manganese, zinc, copper, and nickel may be affected. As macro- and micronutrients are essential for many plant processes and also for pathogen resistance, their undersupply can contribute to the reported toxic effects of glyphosate on plants and lower resistance to pathogens. By this mechanism, plant–microorganism interactions, e.g., nitrogen fixation of leguminous plants, can be influenced too. GBH-treated plants and their products very often contain residues of glyphosate and its main metabolite AMPA, in some cases exceeding the maximum residue levels. Animals and humans consuming these products may thus be affected by residues of glyphosate (and formulants), whose potential toxicity is subject of an ongoing debate. Whether the toxic effects of GBH on non-plant life, described in many papers, could also be linked to the chelating property, has not been extensively studied. The chelating agent glyphosate might also impact soil life by metals that are bound to soil particles. While the compiled data indicate that the occurrence of natural chelators with considerably higher chelating potentials and the high concentrations of potential counterions make a general additional impact by glyphosate for most metals less likely, this may depend on soil characteristics, e.g., on micronutrient supply, on amounts of glyphosate applied, and on the resolution and scale of the analytical approaches. Therefore, research should be undertaken to elucidate the role of glyphosate as a chelating agent, also considering formulants, in particular, as chelation is a nonspecific property potentially affecting many organisms and processes and as glyphosate-based herbicides are the most often applied herbicides worldwide.

## Footnotes

1 Regulation (EC) 1107/2009 and Regulation (EC) 540/2011

2 http://europa.eu/rapid/press-release_MEX-16-2357_de.htm; http://europa.eu/rapid/press-release_MEMO-16-2012_en.htm

3
https://ec.europa.eu/food/sites/food/files/plant/docs/pesticides_glyphosate_commission_proposal_revision2_20171025.pdf

4
http://www1.agric.gov.ab.ca/$department/deptdocs.nsf/all/faq8069

5 http://www.monsanto.com/products/documents/glyphosate-background-materials/rrplus%20i%20-%20the%20science%20of%20roundup%20ready%20technology,%20glyphosate,%20and%20micronutrients%20final%2010-4-11.pdf.

6
http://www.grandviewresearch.com/press-release/global-glyphosate-market

7
https://www.epa.gov/sites/production/files/2015-12/documents/regulations_ccr_ccrtable_0.pdf

8
http://www.lenntech.com/applications/drinking/standards/eu-s-drinking-water-standards.htm

9
http://www.stephanehorel.fr/wp-content/uploads/2016/11/10-CLA_Letter_US_Trade_Rep2013.pdf

10
http://ec.europa.eu/food/plant/pesticides/eu-pesticides-database/public/?event=pesticide.residue.CurrentMRL&language=EN

11 http://www.ecfr.gov/cgi-bin/searchECFR?ob=c&idno=40&q1=glyphosate&r=&SID=9d076cfdbd1419603bcd6c6c06ff6bca&mc=true, https://www.ecfr.gov/cgi-bin/text-idx?SID=e97fe740d0508bfd7d27de5518e7f551&mc=true&node=pt40.26.180&rgn=div5#se40.26.180_1364

12
http://www.enzyme-database.org/query.php?name=phosphoenolpyruvate&search=search_all&display=show_all&order=ec_num&nr=50

13
http://eur-lex.europa.eu/legal-content/EN/ALL/?uri=CELEX:32013R0284
